# Evaluation of a hearing assessment protocol for children with communication or autism concerns

**DOI:** 10.3389/fped.2026.1833679

**Published:** 2026-06-22

**Authors:** Eriel J. Confer, Alyssa M. Fosnight, Lindsey R. Wheeler, Angela Yarnell Bonino

**Affiliations:** 1 Department of Hearing and Speech Sciences, School of Medicine, Vanderbilt University, Nashville, TN, United States; 2 Department of Hearing and Speech Sciences, Vanderbilt University Medical Center, Nashville, TN, United States

**Keywords:** audiology, autism, developmental delay, hearing evaluation, hearing loss, hearing screening, pediatrics

## Abstract

**Introduction:**

Autistic children often face barriers to accessing timely and accurate hearing evaluations. Yet determining a child's hearing status is important for maximizing developmental outcomes when there are communication concerns.

**Methods:**

This study evaluated the effectiveness of a standardized hearing assessment protocol for children aged 18 months to 8 years, with and without an autism diagnosis in their medical record. The protocol aimed to determine whether sufficient hearing data were available for children to proceed with speech-language or autism evaluations (“speech criteria”) and/or be discharged from audiology care (“discharge criteria”).

**Results:**

Retrospective analyses of 608 children's medical records from a single institution revealed that 93.9% of children met speech criteria and 56.6% met discharge criteria. Relative to children in the comparison group, autistic children were significantly less likely to meet speech or discharge criteria (all *p*-values < .05), required nearly twice as many encounters to do so, and relied more heavily on physiological measures (e.g., distortion product otoacoustic emissions and sedated auditory brainstem responses). Despite these differences, many autistic children successfully met criteria by the second encounter, demonstrating the protocol's overall effectiveness.

**Discussion:**

Findings underscore the need for protocol refinement by incorporating tailored supports that address sensory sensitivities, communication differences, and learning profiles of autistic children. Future research should investigate strategies to optimize behavioral testing and design fast-track pathways based on physiological assessments. The protocol presented here has the potential to advance patient care and improve timely and accurate determination of hearing status in children undergoing a speech-language and/or autism evaluation.

## 1 Introduction

It is estimated that 1 in 31 children are on the autism spectrum in the United States (1). Autism spectrum disorder (ASD) is a group of neurodevelopmental conditions characterized by challenges with social skills, communication, and repetitive or restricted behaviors (2). To assist in the differential diagnosis process, a hearing evaluation is recommended (3, 4). Hearing evaluation results provide insights on if (1) undiagnosed reduced hearing is as a contributing factor for potential developmental delays, and (2) a child has sufficient auditory access to oral directions and prompts that are used during standardized developmental assessments. Hearing status needs to be quickly and accurately determined to avoid delays in the assessment process. Moreover, it is important to determine hearing status for autistic children because many genetic and brain-based causes of autism are associated with congenital or late-onset reduced hearing (e.g., prematurity, congenital cytomegalovirus, Down syndrome) (5). However, the hearing assessments that comprise the comprehensive pediatric test battery were not designed for the unique developmental or health needs of autistic children. Consequently, autistic children are vulnerable to receiving delayed or no access to key hearing assessments in the test battery (6, 7). The purpose of this study was to evaluate the effectiveness of a hearing assessment protocol for children aged 18 month to 8 years, with and without an autism diagnosis in their medical record.

For the age range of children in the present study, the gold-standard assessment of hearing is the audiogram (4). The audiogram measures the lowest intensity level at which frequency-specific signals can be detected (i.e., threshold). Behavioral test methods used to collect thresholds rely on the principles of operant conditioning to teach a child to perform a target, motor response when the signal is heard. Behavioral test methods can be used starting at a developmental age of approximately 6 months. These methods employ response behaviors, reinforcement materials, and training paradigms that are based on typical child development. When behavioral testing cannot be successfully performed, hearing status should be estimated using auditory brainstem response (ABR) testing. ABR is a physiological measure of auditory pathway integrity from the cochlea to the brainstem. During ABR testing, a child must remain quiet and still. To achieve this, children in the age range of the present study typically require general anesthetics or sedation drugs (8). However, some children may have health contraindications for sedation, or the medical team may delay ABR testing to combine it with other sedated procedures to reduce the risks of repeated drug exposure (9). To complete a comprehensive hearing evaluation, audiogram or ABR thresholds should be cross-checked with speech audiometry and physiological assessments. Each physiological assessment provides partial information about the auditory system. Specifically, tympanometry assesses middle-ear dysfunction (e.g., otitis media). Otoacoustic emissions (OAEs) measure the function of the outer hair cells in the cochlea. OAEs are not sensitivity to neural forms of differences in hearing (e.g., auditory neuropathy spectrum disorder), and often miss reduced hearing cases that are classified in the “mild” severity level (10). Middle ear muscle reflexes (MEMRs) evaluate the integrity of the auditory pathways by eliciting the stapedial reflex in response to high-intensity sounds. All of these physiological assessments do not require active responses from the child, but they do require the child to remain still, quiet, and tolerate a probe in their ear for up to one minute.

The standard hearing test battery was documented to be feasible and interpretable for autistic children nearly two decades ago (11, 12). These researchers compared performance on a protocol of clinical assessments in a laboratory setting for autistic children to age-matched children with typical development. Relative to children with typical development, results from both studies indicated that autistic children had similar rates of completion and comparable ranges of values for tympanometry, OAEs, MEMRs, and ABR. Additionally, Gravel et al. reported that behavioral thresholds were similar between the two groups of children (6–12 years) (11). In contrast, Tharpe et al. reported that nearly half of the autistic children (3–10 years; *N* = 22) exhibited elevated or unreliable behavioral thresholds despite evidence of typical hearing on physiological assessments (12). Tharpe et al. proposed that elevated thresholds may reflect hyposensitivity to sound or differences in auditory processing. However, an alternative explanation is that unreliable thresholds are the result of misalignment between children's skills and the developmental requirements of the test methods. Behavioral test methods assume uniform development within and across multiple developmental domains. However, autistic children often experience delays or differences in executive function skills (e.g., shifting attention, impulse control), motor planning and coordination, and participating in activities led by unfamiliar people (2). Moreover, differences in cognitive abilities between the two samples may explain differences. Specifically, 86% (19 out of 22) of autistic children tested by Tharpe et al. had cognitive scores below the “average” cutoff score, whereas fewer than 10% (3 out of 27) of children tested by Gravel et al. did. Autistic children, especially those with co-occurring intellectual disability, may demonstrate elevated or unreliable behavioral thresholds due to delays in non-auditory developmental skills that are required to perform behavioral test methods.

Amplifying these challenges, recent retrospective clinical studies reveal access barriers and delays in the definitive diagnosis of hearing status for autistic children (6, 7, 13, 14). By reviewing Military Health System electronic medical records, Meagher et al. found that 56% of 1.5- to 5-year-old, autistic children had no or limited behavioral thresholds collected during their initial hearing evaluation (13). When little or no behavioral information is obtained, ABR testing can serve as an alternative gold-standard assessment. However, recent findings based on retrospective clinical data indicate that ABR testing may not be as accessible as previously assumed for autistic children. Bonino et al. reported that fewer than 2% of autistic children accessed ABR testing during the initial 3-month period of hearing health care at three academic hospitals in the United States (7). Furthermore, relative to children without a developmental disability diagnosis, autistic children were three times less likely to have audiogram or ABR thresholds measured within the first 3 months of hearing health care (7). Additionally, Trudeau et al. reported that the average delay between the first hearing evaluation and sedated ABR testing at their hospital was 9 months for autistic children (6). Delays or failure to collect audiogram or ABR thresholds can postpone the autism evaluation process, or delay access to appropriate deafness-related interventions aimed at mitigating the effects of sensory deprivation due to reduced or absent auditory access (e.g., sign language, hearing technology). These findings highlight the need for a better understanding of how developmental profile influences the selection of diagnostic tests, the prioritization of audiological information, and the clinical decision-making process. Without clear, evidence-based protocols tailored to the needs of autistic children, delays in hearing evaluations may continue to negatively impact developmental outcomes.

To address these gaps and barriers in care, the present study aimed to evaluate the effectiveness of a hearing assessment protocol for children aged 18 months to 8 years with and without an autism diagnosis in their medical record. All children were scheduled for a hearing evaluation prior to a speech-language evaluation. In addition to using a comprehensive test battery approach, the protocol established two criteria for determining whether sufficient hearing data were obtained. The first criterion, referred to as the “speech criteria”, was met when there was sufficient hearing data to confirm that a child has access to aural speech. Meeting the speech criteria allowed children to proceed with their scheduled speech-language evaluation or dual speech-language and autism evaluation. The second criterion, referred to as the “discharge criteria”, was met when there was sufficient evidence to confirm typical hearing abilities in both ears. Meeting the discharge criteria allowed children to be discharged from the audiology clinic, assuming they do not have risk factors for changes in hearing status.

We proposed three research questions for the present study:

(1) *What percentage of children provided sufficient information to meet speech criteria or discharge criteria?* We predicted that, compared to children in the comparison group, autistic children would have lower odds of meeting speech and discharge criteria. Consequently, autistic children were expected to require more encounters to obtain sufficient hearing data to meet criteria.
(2) *What audiological assessments were used to meet speech or discharge criteria?* We anticipated that the utilization rate of specific hearing assessments will differ based on autism classification. Of particular interest is to test the hypothesis that behavioral test methods (i.e., audiogram) were used less frequently for autistic children than for children in the comparison group.
(3) *Are risk factors and the rate of reduced hearing similar between the two groups of children?* We anticipated that the prevalence of risk factors and reduced hearing status would be similar across the two groups.

Results from this study have the potential to advance patient care, as there is currently no standardized hearing assessment protocol for autistic children with documented clinical effectiveness in real-world practice.

## 2 Methods and materials

### 2.1 Ethics statement

This study was determined to be exempt by the Institutional Review Board at Vanderbilt University Medical Center.

### 2.2 Overview of Vanderbilt protocol

**Figure 1:**
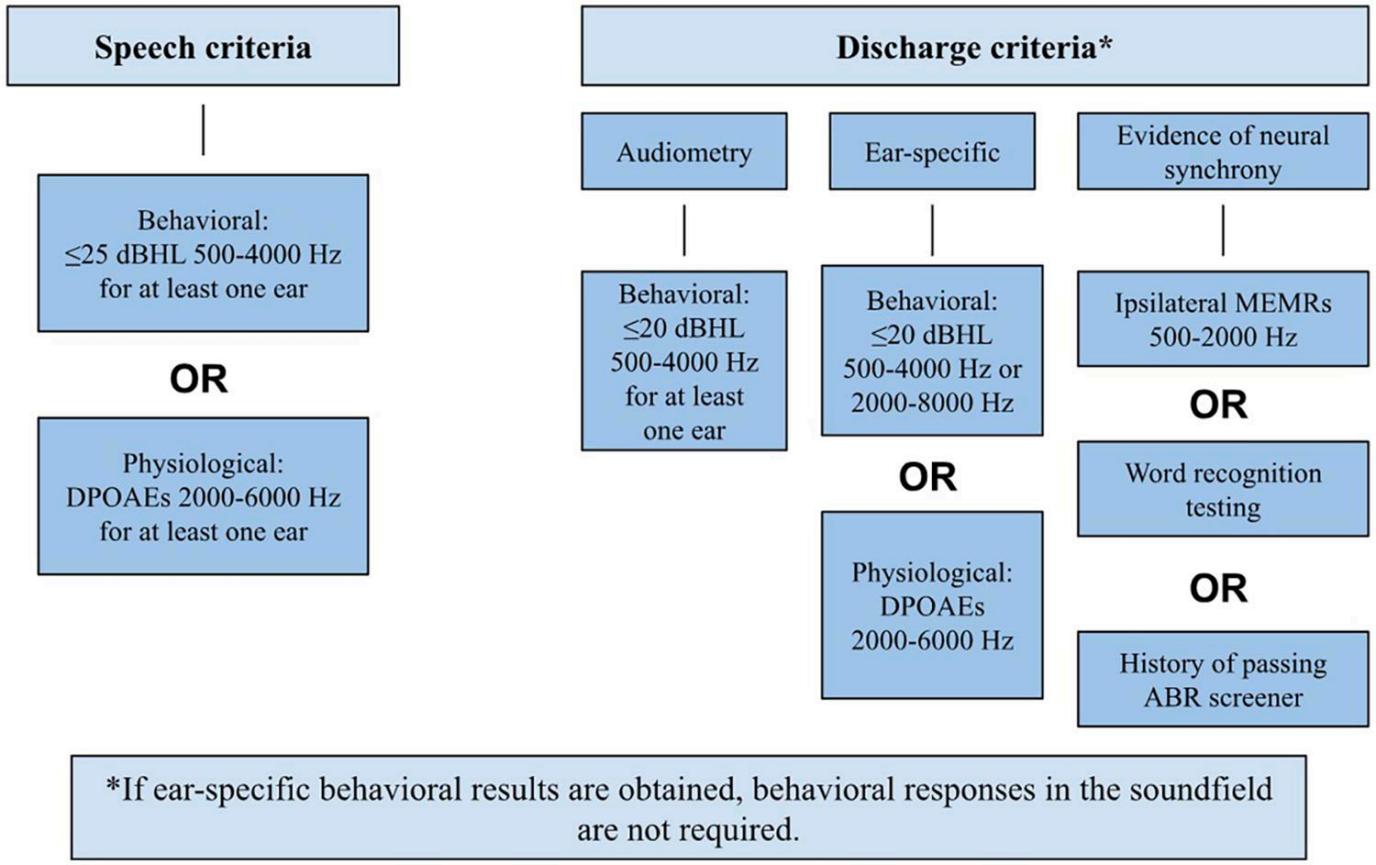
Required assessments to meet speech or discharge criteria. ABR thresholds can be used in lieu of ear-specific audiogram results.

In 2018, a standardized assessment protocol was implemented to evaluate hearing in all children prior to evaluations conducted in the pediatric speech-language clinic at Vanderbilt University Medical Center (VUMC). The speech-language clinic conducts speech-language evaluations, as well as dual speech-language and autism evaluations. Figure 1 provides an overview of the comprehensive protocol, which includes behavioral and physiological measures of hearing. Components of the protocol are discussed in detail below. The protocol provides two pathways to determine whether there is sufficient hearing data to (1) allow the child to proceed with the speech-language evaluation or dual speech-language and autism evaluation, and (2) discharge the child from audiological care because typical hearing status was confirmed.

#### 2.2.1 Components of diagnostic hearing assessment

As is recommended for a comprehensive pediatric hearing evaluation (4, 15), our clinical protocol incorporates both behavioral and physiological measures of hearing. At the core of the protocol is the audiogram. Testing is performed with a behavioral test method that is expected to be developmentally appropriate for the child based on clinician judgement. Visual reinforcement audiometry (VRA) is typically used for children with a developmental age of approximately 6 to 24 months. In VRA, the child is conditioned to turn their head toward a visual reinforcer, such as a video or mechanical toy, when a sound is presented. For children with a developmental age of 2 to 5 years, conditioned play audiometry (CPA) is used. CPA involves teaching the child to perform a motor response, such as placing a peg in a board, when a sound is heard. For children whose developmental abilities fall between those required for VRA and CPA, a hybrid approach may be used. Conventional audiometry is used for older children and requires the patient to verbally indicate, raise their hand, or press a button when a sound is heard. Regardless of the test method, clinicians aim to collect thresholds from 250 to 8,000 Hz for both ears.

When behavioral testing cannot be performed, ABR testing can be used to estimate hearing sensitivity. At a minimum, ABR thresholds are measured for tone-burst stimuli at frequencies ranging from 500 to 4,000 Hz for each ear. For children in the age range of the present study, ABR testing typically requires sedation. At our institution, ABR testing can be performed under general anesthesia in the operating room, or with moderate sedation (e.g., intranasal dexmedetomidine, propofol, and/or ketamine) in a sedation suite adjacent to the operating room with monitoring by an anesthesiologist. ABR testing performed in the operating room may be conducted in isolation or combined with other sedated procedures [e.g., placement of pressure equalization (PE) tubes, dental work].

To cross-check audiogram or ABR thresholds, speech audiometry and physiological measures of hearing are performed. Speech audiometry includes speech recognition thresholds or word recognition in quiet (open- or closed-set). Three physiological measures are used in the protocol, all of which provide partial information about the state of the auditory system. Tympanometry is used to assess middle ear function (e.g., the presence of fluid or patency of PE tubes). Probe tones of 226 Hz or 1,000 Hz are used depending on the child's age, and results are classified according to the Jerger system. Distortion product otoacoustic emissions (DP OAEs) are measured from 2,000 to 6,000 Hz to evaluate the function of outer hair cells in the cochlea. To be considered “present”, DP OAE responses must meet a two-step criterion at all frequencies: (1) amplitude > 0 dB SPL, and (2) a signal-to-noise ratio of ≥6 dB (16). This reflects the minimum DP OAE protocol required; clinicians may elect to collect responses from additional frequencies. Ipsilateral MEMR thresholds are typically obtained from 500 to 2,000 Hz, with contralateral thresholds collected as indicated. Collectively, a combination of behavioral and physiological assessments comprised the comprehensive test battery used to determine hearing status.

#### 2.2.2 Requirements for meeting speech criteria

Speech criteria are the minimum requirements for hearing data that allow a child to proceed with their speech-language evaluation. Satisfactory requirements include either (1) behavioral hearing thresholds of ≤25 dB HL at 500, 1,000, 2,000, and 4,000 Hz or (2) present DP OAEs from 2,000 to 6,000 Hz. Data are only required for one ear, and soundfield thresholds are permitted. To classify as meeting speech criteria, required data must be obtained prior to the speech-language evaluation. Only hearing data collected the 12 months prior to the speech-language evaluation were reviewed. Data collected after the speech-language evaluation were not used to meet speech criteria.

#### 2.2.3 Requirements for meeting discharge criteria

To release a child from audiology care, both typical peripheral functioning and neural integrity of the auditory system must be documented. To meet discharge criteria, either audiogram or ABR thresholds (from 500 to 4,000 Hz) are required. Audiogram thresholds must be ≤20 dB HL, and ABR thresholds must be ≤20 dB eHL. If ear-specific audiogram thresholds are not obtained, present DP OAEs (2,000 to 6,000 Hz) must be documented for both ears in conjunction with audiogram thresholds in the soundfield. To establish neural integrity, one or more of the following results must be documented: present MEMRs at 90 to 95 dB SPL (at 500, 1,000, and 2,000 Hz), word recognition scores that are consistent with behavioral thresholds, or a history of passing an ABR newborn hearing screening (17–19). In the present study, word recognition scores of ≥80% were confirmed to meet discharge criteria. To classify as meeting discharge criteria, required data had to be obtained within 12 months of the speech-language evaluation. Data collected after the speech-language evaluation were permitted. For children with risk factors for changes in hearing, additional monitoring may be recommended which will delay or prevent discharge.

#### 2.2.4 Implementation considerations

This protocol was established to ensure that adequate hearing data are available prior to a speech-language or autism evaluation. Audiologists across all 5 VUMC pediatric audiology sites are encouraged to follow this standardized diagnostic protocol. Children are routinely scheduled for a 45 min evaluation, whereas children seen in conjunction with otolaryngology care are scheduled for a 30 min hearing evaluation slot. Modifications to the protocol may be made based on child factors, or site-specific constraints. For example, audiologists modify assessment type or procedures to accommodate the child's developmental profile, medical status, or prior hearing data. A child may be discharged with a 25 dB HL threshold at 500 Hz because of PE tubes. The protocol may be modified when there are schedule constraints (e.g., patient arrived late to the clinic). A final example is that additional testing may be scheduled at the request of otolaryngologists—even if discharge criteria are met—to monitor hearing before or after medical interventions.

Pediatric audiologists at VUMC are highly familiar with autism. They have completed training on common features of autism; they routinely administer the Modified Checklist for Autism in Toddlers, Revised (MCHAT-R) to screen for autism (20). If autistic traits are observed and an evaluation is not scheduled, audiologists are encouraged to discuss options for autism evaluation and possible referral pathways with families. For children who are scheduled for a speech-language evaluation at VUMC, this appointment can be modified to a dual evaluation, assessing both speech-language and autism. Whereas a developmental work-up with VUMC's developmental medicine team can be recommended for children with complex medical and/or developmental profiles (e.g., Down syndrome, history of trauma), or who speak languages other than English, Spanish, or Arabic. For children under 3 years, additional expedited referral pathways are available at VUMC, including a tele-health autism assessment option.

### 2.3 Construction of dataset

For the present study, retrospective analyses were performed on medical records from patients who underwent a speech-language evaluation at VUMC between October 1, 2023, and October 31, 2024. We restricted the child's age at the time of the speech-language evaluation to ≥18 months and <8 years. This age range was chosen to align with the age requirements for obtaining an autism evaluation in conjunction with a speech-language evaluation. Only children whose preferred languages were English, Arabic, or Spanish were included in the dataset, as dual speech-language and autism assessments are only available in these three languages. We excluded children with craniofacial abnormalities (including Down syndrome and cleft lip/palate) or with ear malformations. Children were identified with these conditions based on the International Classification of Diseases, 10th Revision (ICD-10) codes or information in the audiological report. The rationale for excluding children with these structural differences is that they require more intensive audiological monitoring than outlined in the current protocol (21–23). Additionally, we excluded children who had known permanent reduced hearing at the time of scheduling their speech-language evaluation.

To construct the dataset, we integrated data from three sources: the Research Derivative, a manual chart review of audiology and speech-language encounter notes, and AudBase. The Research Derivative is a database of clinical and related data derived from VUMC's clinical systems (e.g., Epic) for research use. First, the Research Derivative was used to extract medical record numbers for all patients who had the CPT code 92523 during the specified time span. The CPT code 92523 is used in our department for children who are receiving either a speech-language evaluation or a dual speech-language and autism evaluation. We obtained demographic and clinic history data for identified children from the Research Derivative. These variables included medical record numbers, ICD-10 diagnoses, zip code, insurance status, and encounter history for the audiology or SLP clinics. Next, a manual chart review was completed for children identified by the Research Derivative pull. Patients who were seen for a speech-language evaluation without completing a hearing evaluation were excluded (*n* = 258). We examined any hearing evaluation in the child's record that was completed within 12 months of the speech-language evaluation. As part of the chart review, we coded the type of assessments used, hearing status, and the clinician's recommendations. Additionally, we coded for risk factors associated with reduced hearing in childhood based on the Joint Commission on Infant Hearing's (JCIH) guidelines (5). Due to variability in clinician documentation practices, the research team used best judgment based on the audiology notes in the child's record. Additionally, we extracted audiological results from AudBase for children in the dataset. AudBase is a software system that collects and stores audiology data electronically. At VUMC, AudBase is used to capture raw behavioral threshold, tympanometry, MEMR, and speech audiometry data.

### 2.4 Patient demographics

Patients were classified based on age, sex, race and ethnicity, insurance type, and level of neighborhood resources. Age was defined as the child's age in years at the time of the encounter. Sex was coded based on biological characteristics as classified in the electronic medical record. Race was categorized into 7 groups, mirroring available options in VUMC's electronic health record system: Asian, Black/African American, Hispanic/Latino, Middle Eastern or Northern African (MENA), Other, Two or More Races, or White. Native American, Pacific Islander and Native Hawaiian were available categories in the system but were not selected for any children in the dataset. Hispanic/Latino could be recorded under race or ethnicity. Because of this overlap and the high rate of missing ethnicity data, these variables should be interpreted cautiously. The electronic medical record included the type of insurance the patient had. A list of these insurances was compiled and classified as either public or private insurance.

Lastly, due to various disparities that affect patients' access to care, the extent of resources available to a patient was estimated using the Child Opportunity Index (COI, version 3.0-2023). The COI provides an estimate of neighborhood-level resources linked to health care access and outcomes (24, 25). The COI is a composite index of 44 components across three domains: education, health and environment, and social and economic factors. We used the zip code listed in patients' records at the time of the speech-language evaluation and extracted COI values based on 2023 data. The COI classification includes five categorical levels of resources in neighborhoods based on national scores: “Very Low” (COI score in the ≤20th percentile for zip codes in the United States), “Low” (21–40th percentile), “Moderate” (41–60th percentile), “High” (61–80th percentile), and “Very High” (>80th percentile).

### 2.5 Autism classification

Children were identified as having an autism diagnosis if they had at least one entry of the ICD-10 diagnosis code of F84.0 in their electronic medical record. The code could have been recorded at any time point and by any provider at VUMC. An autism diagnosis was not required to be documented at the time of the speech-language or the hearing evaluation. Children who did not have the ICD-10 diagnosis code of F84.0 in their records were placed into the comparison group for the present study.

### 2.6 Statistical procedures

Before conducting analyses, we used data-cleaning techniques to organize and extract data across multiple.csv files. This process was performed in Python (version 3.9) using a JupyterLab notebook (version 3.4). Descriptive statistics and regression models were conducted using IBM SPSS Statistics (version 29.0). First, unadjusted odds ratios (ORs) were computed from binary regression models to examine potential differences in demographic characteristics based on group membership (i.e., autistic vs. comparison group). *T*-tests were conducted to examine potential age-related differences for points in clinical care based on group. Next, binomial regression models were used to test the main effects of group (0 = “Comparison”, 1 = “Autism”) on the likelihood of meeting speech criteria or discharge criteria (0 = “No”, 1 = “Yes”). Separate binomial regression models were constructed to examine the likelihood of having results on a specific assessment type (0 = “No”, 1 = “Yes”) based on group (0 = “Comparison”, 1 = “Autism”). Additionally, generalized linear models (GLMs) with a Poisson link function were constructed to examine the number of required encounters to meet speech or discharge criteria based on group (0 = “Comparison”, 1 = “Autism”). Lastly, we examined whether the likelihood of having at least JCIH risk factor recorded (0 = “No”, 1 = “Yes”) differed based on group (0 = “Comparison”, 1 = “Autism”). Except for the initial unadjusted ORs reported for demographic factors, all other models accounted for both child age at the time of the initial hearing evaluation (in years) and COI (Very Low, Low, Moderate, High, and Very High). COI was included as a covariate because it provides a multidimensional composite of neighborhood-level opportunity, whereas insurance type if frequently confounded by disability status. All analyses were conducted at an alpha level of 0.05 for significance. ORs with 95% confidence intervals (CIs) are reported for these models.

## 3 Results

### 3.1 Overview of patients

The final dataset was 608 children. Nearly 40% of children (*n* = 227) in the dataset had at least one entry of an autism diagnosis in their medical record. An autism diagnosis was received by 154 children as the result of a dual speech-language and autism evaluation in our department. Table 1 provides an overview of demographic variables for children stratified by autism classification. There were no statistically significant differences in sex, race, preferred language, or COI based on autism classification. However, there was a significant difference in insurance type between the two groups. A higher proportion of autistic children used public insurance compared to children in the comparison group [*p* = 0.007; OR = 1.80; 95% CI: (1.17, 2.75)].

**Table 1:** Characteristics of children by autism classification. Percentages represent the proportion of children for each demographic characteristic within each autism classification group. Unadjusted odds ratios (OR) were calculated to assess differences in the distribution of specific demographic characteristics between autistic children and children in the comparison group.

Demographics	*N*	Autism classification		OR	*p*	95% CI
		Yes *n* (%)	No *n* (%)			
Total	608	227 (37.3%)	381 (62.7%)	—	—	—
Sex						
Female	196	64 (28.2%)	132 (34.6%)	.74	.100	.52, 1.06
Male^d^	412	163 (71.8%)	249 (65.4%)	—	—	—
Race^a^						
Asian	6	4 (1.9%)	2 (.6%)	3.81	.128	.68, 21.29
Black/African American	166	67 (31.8%)	99 (27.7%)	1.29	.234	.85, 1.96
Hispanic/Latino	124	43 (20.4%)	81 (22.7%)	1.01	.961	.64, 1.61
Middle Eastern/North African	11	2 (.9%)	9 (2.5%)	.42	.280	.09, 2.01
Other	6	4 (1.9%)	2 (.6%)	3.81	.128	.68, 21.29
Two or More Races	40	17 (8.1%)	23 (6.4%)	1.41	.329	.71, 2.80
White^d^	215	74 (35.1%)	141 (39.5%)	—	—	—
Ethnicity^b^						
Hispanic/Latino	177	65 (98.5%)	112 (95.6%)	—	—	—
Non-Hispanic	5	1 (1.5%)	4 (3.4%)	—	—	—
Preferred language						
Arabic	16	5 (2.2%)	11 (2.9%)	.75	.590	.25, 2.18
English^d^	459	174 (76.7%)	285 (74.8%)	—	—	—
Spanish	133	48 (21.1%)	85 (22.3%)	.93	.703	.62, 1.38
Insurance^d^						
Private^d^	130	38 (18.0%)	92 (28.3%)	—	—	—
Public	406	173 (82.0%)	233 (71.7%)	1.80	.007	1.17, 2.75
Child opportunity index						
Very Low^d^	62	22 (9.7%)	40 (10.5%)	—	—	—
Low	240	96 (42.3%)	144 (37.8%)	1.21	.516	.68, 2.17
Moderate	138	48 (21.1%)	90 (23.6%)	.97	.923	.52, 1.82
High	127	48 (21.1%)	79 (20.7%)	1.11	.757	.59, 2.08
Very High	41	13 (5.7%)	28 (7.3%)	.84	.692	.37, 1.95

a Missing = 40 (6.6%).

b Missing = 426 (70.1%); OR not examined due to high rate of missing cases.

c Missing = 72 (11.8%).

d Referent category for logistic regression.

OR, odds ratio (unadjusted); CI, confidence interval.

Table 2 reports the observed distribution of child age at five time points: first hearing evaluation, speech-language evaluation, autism diagnosis (if applicable), meeting speech criteria, and meeting discharge criteria. Recall that the first hearing evaluation refers only to encounters reviewed within the 12 months prior to the speech-language evaluation. A total of 58 children had audiology encounters preceding the review period, and therefore, those encounters were excluded from the present study (*n* = 18 autistic children). Age at the time of the speech-language evaluation (*p* = .005; mean difference = 3.3 months) and age at meeting discharge criteria (*p* = .025; mean difference = 4.1 months) were significantly older for autistic children relative to the comparison group. In contrast, age at the time of the first hearing evaluation (*p* = .082) and age at meeting speech criteria (*p* = .085) were similar between the two groups. Due to these age-related differences, child age was accounted for in all subsequent analyses. Age at the first hearing evaluation was used as a covariate in all regression models, as this reflects the first step in determining hearing status.

**Table 2:** Summary of age characteristics (in years) for children based on autism classification. Ages are reported at several time points, with the median and interquartile range (25th and 75th percentiles) provided for each group. Two-tailed *t*-tests were performed to assess whether child age differed at each clinical step based on group.

		Autism classification			*T*-test
	*N*	Yes Median age (IQR)	No Median age (IQR)	*p*	Mean Difference [95% CI]
Age at first hearing evaluation	608	2.72 (2.08, 3.59)	2.55 (1.96, 3.30)	.082	.17 [−.02,.37]
Age at speech-language evaluation	608	2.99 (2.33, 3.86)	2.70 (2.08, 3.44)	.005	.27 [.08,.46]^a^
Age at autism diagnosis	227	2.82 (2.27, 3.63)	–––	–––	–––
Age at meeting speech criteria	571	2.82 (2.06, 3.70)	2.58 (2.01, 3.33)	.085	.18 [−.02,.38]
Age at meeting discharge criteria	344	3.32 (2.34, 4.06)	2.90 (2.21, 3.66)	.025	.34 [.04,.64]^b^

a Cohen's *d* = .24, small effect.

b Cohen's *d* = .27, small effect.

### 3.2 Meeting speech criteria

Out of the 608 children in the dataset, 571 children (93.9%) met speech criteria. A binomial regression model was constructed to examine the likelihood of meeting speech criteria based on autism classification while accounting for child age and COI. Based on this model, autistic children were significantly less likely to have sufficient data to meet speech criteria compared to children in the comparison group [90.7% vs. 95.8%; *p* = .009; OR = .40; 95% CI: (.20,.80)].

Next, we examined data only from children who successfully met speech criteria. Table 3 reports the types of assessments used to successfully meet speech criteria stratified by group. Separate models were constructed for each assessment type. Relative to the comparison group, autistic children had lower odds of having either soundfield thresholds [*p* = .002; OR = .56; 95% CI: (.39,.80)] or ear-specific behavioral thresholds [*p* < .001; OR = .17; 95% CI: (.09,.30)]. The proportion of children evaluated with DP OAEs in only one ear was similar across the two groups (*p* = 0.809). In contrast, autistic children had greater odds of having bilateral DP OAE results [*p* = .007; OR = 1.67; 95% CI: (1.15, 2.42)]. Sedated ABR testing was seldom used to meet speech criteria (*n* = 8). However, autistic children appear to have greater odds of being evaluated with sedated ABR testing than children in the comparison group [*p* = .018; OR = 12.79; 95% CI: (1.55, 105.75)]. These results should be interpreted with caution, as the OR is based on a very limited number of cases, compromising the reliability and precision of the estimate. Given this limitation, the ABR finding should be considered exploratory and warrant replication in larger, more robust datasets to confirm the observed trend. In summary, the results across the different assessment types suggest a higher reliance on physiological measures rather than behavioral testing in autistic children to meet criteria.

**Table 3:** Summary of the number of children who met speech criteria. For the subset of children who met criteria (*n* = 571), the total number of children who underwent each assessment type or required a specific number of encounters is reported. Percentages represent the proportion for the variable within each group. ORs from binomial regression models are reported to evaluate the likelihood of meeting speech criteria or using a specific assessment type, based on autism classification while accounting for child age and COI.

Speech criteria	N	Autism classification		OR	*p*	95% CI
		Yes *n* (%)	No *n* (%)			
Meeting Speech Criteria	571	206 (90.7%)	365 (95.8%)	.40	.009	.20, .80
Type of assessment used to meet criteria						
≤25 dB HL in soundfield	254	72 (35.0%)	182 (49.9%)	.56	.002	.39, .80
≤25 dB HL for both ears	153	33 (16.0%)	120 (32.9%)	.17	<.001	.09, .30
DP OAE for one ear	86	29 (14.1%)	57 (15.6%)	.94	.809	.58, 1.54
DP OAE for both ears	303	121 (58.7%)	182 (49.9%)	1.67	.007	1.15, 2.42
ABR (≤25 dB eHL)	8	7 (3.4%)	1 (.3%)	12.79	.018	1.55, 105.75
Number of encounters required						
1	523	182 (88.3%)	341 (93.4%)	—	—	—
2	35	17 (8.3%)	18 (4.9%)	—	—	—
3	10	6 (2.9%)	4 (1.1%)	—	—	—
4	2	1 (.5%)	1 (.3%)	—	—	—
5	1	0	1 (.3%)	—	—	—

OR, odds ratio adjusted for child age at time of first audiology encounter and COI; CI, confidence interval.

Next, we examined the number of encounters that were required to meet speech criteria. As reported in Table 3, 91.6% of children (*n* = 523) who successfully met speech criteria did so in a single encounter. Of the 48 children who required two or more encounters to meet criteria, 24 (50%) were autistic. To assess this trend, we built a GLM with Poisson link function for the number of encounters based on autism classification, child age, and COI. Only data from the 571 children who met speech criteria were used. The estimated coefficient for a variable in the Poisson GLM can be interpreted as the difference in the logs of expected counts given a one-unit increase in the variable, assuming all other variables are held constant. Thus, the number of encounters required to meet speech criteria increased by a factor of 1.77 [*p* = .023; 95% CI: (1.08, 2.88)] for autistic children diagnosed relative to children in the comparison group, while controlling for child age and COI.

Lastly, for the 37 children who did not meet speech criteria, we examined the reasons why. During the chart review, four reasons were identified and coded: (1) audiologists recommended deferring additional testing and proceeding with the speech-language evaluation (*n* = 3); (2) evidence of reduced hearing was documented (*n* = 2); (3) hearing information was inconclusive (*n* = 24); and (4) medical management for middle-ear dysfunction was required (*n* = 8).

### 3.3 Meeting discharge criteria

Out of our total sample, 344 children (56.6%) had sufficient hearing data in their medical records to meet discharge criteria as defined by the protocol. A binomial regression model was built to examine the likelihood of meeting discharge criteria based on autism classification while accounting for child age and COI. Based on this model, autistic children were significantly less likely to have sufficient data to meet discharge criteria compared to children in the comparison group [41.0% vs. 65.9%; *p* < .001; OR = .32; 95% CI: (.22,.45)]. Table 4 reports the type of assessments used to meet discharge criteria by autism classification. Relative to children in the comparison group, autistic children had lower odds of having ear-specific behavioral thresholds for both ears [*p* = .011; OR = .42; 95% CI: (.21,.82)]. In contrast, autistic children had greater odds of undergoing sedated ABR testing [*p* = .004; OR = 11.28; 95% CI: (2.19, 58.00)]. However, due to the limited number of children undergoing sedated ABR testing, the reported OR should be interpreted cautiously. The use rate of bilateral DP OAEs and specific tests for neural integrity were similar across the two groups (all *p*-values > .05). Thus, these results indicate group differences in the type of assessment used to obtain ear-specific information among children who met discharge criteria.

**Table 4:** Summary of the number of children who met discharge criteria. For the subset of children who met criteria (*n* = 344), the type of assessment used for each requirement and the number of encounters required are reported. Percentages represent the proportion for the variable within each group. ORs from binomial regression models are reported to evaluate the likelihood of meeting discharge criteria or using a specific assessment type, based on autism classification while accounting for child age and COI.

Discharge criteria	*N*	Autism classification		OR	*p*	95% CI
		Yes *n* (%)	No *n* (%)			
Meeting Discharge Criteria	344	93 (41.0%)	251 (65.9%)	.32	<.001	.22, .45
Type of assessment used to meet criteria						
*Not ear-specific audiometry*						
* *≤20 dB HL in soundfield	212	57 (61.3%)	155 (61.8%)	1.57	.179	.81, 3.02
*Ear-specific data*						
* *≤20 dB HL for both ears	152	37 (39.8%)	115 (45.8%)	.42	.011	.21, .82
DP OAE for both ears	204	55 (59.1%)	149 (59.4%)	1.45	.238	.78, 2.68
*Test of neural integrity*						
* *History of passing ABR screening	270	70 (75.3%)	200 (79.7%)	.88	.679	.49, 1.59
* *Present ipsilateral MEMRs	69	15 (16.1%)	54 (21.5%)	.73	.344	.39, 1.39
* *Word recognition	38	9 (9.7%)	29 (11.6%)	.51	.165	.20, 1.32
* *ABR (≤20 dB eHL)	9	7 (7.5%)	2 (.8%)	11.28	.004	2.19, 58.00
Number of encounters required						
1	262	62 (67.4%)	200 (79.7%)			
2	60	18 (19.6%)	42 (16.7%)			
3	17	10 (10.9%)	7 (2.8%)			
4	3	2 (2.2%)	1 (.4%)			
5	1	0	1 (.4%)			

OR, odds ratio adjusted for child age at time of first audiology encounter and COI; CI, confidence interval.

Next, we examined group differences for the number of encounters required to meet discharge criteria. To assess this trend, we fit a GLM with Poisson link function using the same approach as earlier (*n* = 344 children). Based on the estimated coefficient from the GLM, the number of encounters required to meet discharge criteria increased by a factor of 1.97 [*p* < .001; 95% CI: (1.33, 2.91)] for autistic children relative to the comparison group, while controlling for child age and COI.

Lastly, Table 5 reports the reasons coded by study personnel for the 308 children who were delayed (i.e., required 2 or more encounters) or failed to meet discharge criteria. The most common reason was cancelation, rescheduling, or not showing for an appointment (*n* = 108). Additionally, 96 children only had partial hearing information available in their medical record as they did not complete all required assessments in the protocol. No child was coded as having an unstable or complex medical profile as the reason for not meeting discharge criteria. Only two of the reasons were statistically significant based on autism classification. First, relative to the comparison group, autistic children had nearly three times the odds of not learning the behavioral test method as the reason coded for delays or not meeting discharge criteria [*p* = .039; OR = 2.94; 95% CI: (1.06, 8.17)]. Second, autistic children were less likely to need monitoring for possible late-onset reduced hearing compared to children in the comparison group [*p* = .027; OR = .10; 95% CI: (.01,.76)]. These results should be interpreted cautiously, as the limited frequency of certain codes may affect these estimates.

**Table 5:** Summary of potential reasons recorded for 308 children in the dataset who experienced a delay (i.e., required 2 or more encounters) or failed to meet discharge criteria. Only one reason was recorded per child. .

Reason	*N*	Autism classification		OR	*p*	95% CI
		Yes *n* (%)	No *n* (%)			
Medical management for middle-ear dysfunction	37	15 (10.6%)	22 (13.3%)	.71	.355	.35, 1.46
Not able to condition for behavioral testing	19	13 (9.2%)	6 (3.6%)	2.94	.039	1.06, 8.17
Cancelation or rescheduling issues	108	52 (36.6%)	56 (33.7%)	1.09	.733	.67, 1.75
Incomplete or partial hearing info	96	47 (33.1%)	49 (29.5%)	1.29	.317	.78, 2.13
Retest date had not occurred at the time of coding	20	10 (7.0%)	10 (6.0%)	1.23	.661	.49, 3.12
Reduced hearing identified	14	4 (2.8%)	10 (6.0%)	.47	.229	.14, 1.61
Risk factor for reduced hearing (continued monitoring needed)	14	1 (.7%)	13 (7.8%)	.10	.027	.01, .76

OR, odds ratio adjusted for child age at time of first audiology encounter and COI; CI, confidence interval.

Among the reasons for delayed discharge or failure to meet discharge criteria, it is noteworthy that no significant difference was observed in the occurrence of middle-ear dysfunction (*p* = .355) or reduced hearing (*p* = .229) based on autism classification. In total, 37 children were identified with middle-ear dysfunction requiring medical management. An additional 14 children were found to have reduced hearing. Of these 14 children, 3 had reduced hearing that was sensorineural, including 2 autistic children.

### 3.4 Occurrence of JCIH risk factors

Of the 603 children with available history information in their audiological reports, 94 children (15.6%) had at least one JCIH risk factor for reduced hearing in childhood. The occurrence rates of these factors in the dataset are shown in Table 6. The occurrence rate of having at least one JCIH risk factor was similar for the two groups: 14.2% for the group of autistic children and 16.4% for the comparison group [*p* = .470; OR = .84; 95% CI: (.53, 1.34)]. Given the low occurrence rate for individual JCIH risk factors, formal statistical analyses were not conducted to examine differences based on autism classification.

**Table 6:** Characteristics of JCIH risk factors for children by autism classification. Risk factors were determined based on the history in the audiological report. Sufficient history information was available for 603 children (*n* = 226 autistic children). Reported percentages reflect the prevalence of risk factor within each autism classification group.

Risk factors	*N*	Autism classification	
		Yes *n* (%)	No *n* (%)
None	509	194 (85.9%)	315 (83.6%)
One risk factor	82	29 (12.8%)	53 (14.0%)
Two or more risk factors	12	3 (1.3%)	9 (2.4%)
Specific risk factor			
Family history	37	17 (7.5%)	20 (5.3%)
NICU stay > 5 days	55	14 (6.2%)	41 (10.9%)
Aminoglycoside administration > 5 days	5	1 (.4%)	4 (1.1%)
Asphyxia or HIE by 9 months	5	0 (0%)	5 (1.3%)
Culture-positive infections associated with reduced hearing	2	1 (.4%)	1 (.3%)
Significant head trauma or chemotherapy	2	1 (.4%)	1 (.3%)
Genetic syndrome associated with reduced hearing	2	1 (.4%)	1 (.3%)

The most common risk factor reported in our sample was an extended NICU stay of greater than 5 days (*n* = 55). Thirty-seven children had a family history of early, late, or progressive onset of reduced hearing in childhood. Five children had a positive history of receiving aminoglycosides for more than 5 consecutive days. Other less frequently recorded factors included asphyxia or hypoxic-ischemic encephalopathy (HIE; *n* = 5); culture-positive infections (*n* = 2); significant head trauma or chemotherapy (*n* = 2); and genetic syndromes associated with reduced hearing (*n* = 2). Based on the audiological report, no children in the dataset were identified with hyperbilirubinemia with exchange transfusion, extracorporeal membrane oxygenation (ECMO), or *in utero* infections associated with reduced hearing (e.g., cytomegalovirus). Caregiver concern was not evaluated in the present study because it was inconsistently reported in the history despite children being scheduled for a speech-language evaluation. Of the 3 children documented to have reduced hearing that was sensorineural, only one child had a JCIH risk factor.

## 4 Discussion

This study evaluated the clinical feasibility and effectiveness of a standardized hearing assessment protocol at a single institution for children aged 18 months to 8 years based on autism classification. Results from this study demonstrated high overall success rates in meeting speech criteria to proceed with a speech-language evaluation (93.9%) and moderate success rates in meeting discharge criteria (56.6%). Findings from the present study confirm that this clinical protocol is feasible and effective, but there are important differences in testing pathways, success rates, and barriers to care based on autism classification. Relative to the comparison group, autistic children were significantly less likely to meet speech or discharge criteria and required nearly twice as many encounters to do so. Additionally, autistic children were more likely to have their hearing status determined based on physiological measures (e.g., DP OAEs and ABR), whereas children in the comparison group were more likely to complete behavioral testing. These findings provide valuable insights into how the assessment protocol can be tailored to meet the unique needs of autistic children, thereby promoting timely and accurate diagnosis of reduced hearing.

### 4.1 Effectiveness of the hearing assessment protocol

Pediatric guidelines recommend a comprehensive hearing evaluation to accurately determine hearing status. To achieve this objective for children aged 18 months to 8 years, the protocol used in the present study included behavioral audiometry cross-checked with physiological measures and speech audiometry. Laboratory studies have confirmed that this comprehensive test battery is both feasible and clinically interpretable in autistic children, except for elevated behavioral thresholds for some children (11, 12). Results from the present study indicate that only 27.3% of autistic children had sufficient hearing data to meet discharge criteria in a single encounter. For reference, 52.5% of children in the comparison group met discharge criteria in a single encounter. This disparity highlights a critical challenge in clinical practice: initial evaluations often yield limited diagnostic information, contributing to diagnostic uncertainty or delays in assessing hearing status, and an increased risk of loss to follow-up. Moreover, uncertainty and delays in determining hearing status may introduce delays in accessing a speech-language or autism evaluation. These findings underscore the pressing need to improve clinical efficiency of obtaining behavioral and objective assessment information during the first audiologic encounter.

One strategy to improve clinical efficiency—central to the present protocol—is prioritizing the collection of specific types of hearing information to determine whether hearing abilities are sufficient to proceed with speech-language or autism assessments. Results from the present study support this approach: 80.2% of autistic children had sufficient data to meet speech criteria in a single encounter, and another 7.5% by the second encounter. These findings demonstrate the overall effectiveness and flexibility of the speech criteria established in the present protocol.

The flexibility of the permitted assessments within the clinical protocol contributed to children meeting the speech or discharge criteria. Consistent with previous studies (6, 7, 13), we found that autistic children were less likely to provide information through behavioral test methods compared to children in the comparison group. Instead, autistic children were more likely to meet criteria using physiological measures. To meet speech criteria, autistic children had 1.7 times greater odds of using bilateral DP OAEs, and 13 times greater odds of using ABR testing. To meet discharge criteria, autistic children had 11 times greater odds of using sedated ABR testing. However, recall that ABR testing was infrequently used to determine hearing status within the dataset (7.5% of autistic children), and therefore, this estimate should be interpreted cautiously.

These findings warrant further discussion regarding the practical benefits and limitations of relying on physiological measures. Physiological measures are often assumed to be accessible for autistic children, as they do not require active responses from the child. However, successful completion of DP OAEs requires the child to remain quiet and tolerate an ear probe for up to a minute—tasks that may be challenging due to sensory sensitivities, heightened anxiety with an unfamiliar adult, and communication differences common in this population. Sedated ABR may not be accessible to all children due to factors such as cost, sedation risks, or geographic location. Furthermore, depending on testing parameters, these measures may have limited sensitivity to detecting mild losses or atypical configurations across frequencies, or neural-based differences in the case of DP OAEs (10, 17, 26, 27). The reliance on physiological measures may stem from various factors, including challenges in conditioning autistic children for behavioral tasks, clinician familiarity and comfort with objective test methods, or caregiver preference to obtain information quickly and not have multiple encounters. Further research is needed to determine efficacious DP OAE and sedated ABR protocols for identifying reduced hearing in autistic children given that these methods are routinely used in the clinic.

In addition to differences in test methods, autistic children required nearly twice as many encounters to meet screening and discharge criteria compared to children in the comparison group, after adjusting for age. Despite these differences, the overwhelming majority of autistic children (90.7%) ultimately met criteria to proceed with their speech-language evaluations. Therefore, although there was a statistically significant difference in the yield rate for meeting speech criteria based on autism classification, this difference may reflect the increased statistical power provided by the sample size rather than clinically meaningful disparities. Thus, despite differences in testing pathways, these findings indicate that this protocol can effectively be used to determine if there is adequate access to auditory information to proceed with a speech-language or autism evaluation.

In the present study, 2.3% of children were identified with reduced hearing as part of a routine hearing evaluation before their speech-language evaluation. Moreover, the likelihood of identifying reduced hearing was similar across the two groups (autism vs. comparison). While there are mixed results in the literature on the prevalence of co-occurrence of autism and reduced hearing, a systematic review did not find conclusive evidence that autistic children had a higher rate of co-occurring reduced hearing than seen in the general population (28). Similarly, Mishaal et al. reported a 3.3% prevalence rate for reduced hearing among children referred for an autism evaluation (*n* = 4,213 children; 0 to 18 years) (29). In that study, nearly all autistic children with co-occurring reduced hearing either had known permanent reduced hearing or a risk factor for reduced hearing at the time of the autism evaluation. In contrast, of the 3 children identified with reduced hearing that was sensorineural in the present study, only one child had a JCIH risk factor. Our results support the current recommendation for a hearing evaluation for children with communication concerns, regardless of JCIH risk factors or newborn hearing screening results.

In summary, the current protocol effectively determined hearing status for most children referred for a speech-language evaluation or dual speech-language and autism evaluation. While autistic children often require more encounters, the high rate of successful data collection by the second appointment demonstrates that accurate testing is achievable. These findings also prompt reconsideration of what accessibility entails for this population. For autistic children, accessibility must account for sensory sensitivities, communication differences, anxiety, and the need for individualized pacing and teaching strategies. Creating accessible hearing evaluations for autistic children may require adapting not only the clinical environment but also expectations, workflows, and tools. With continued refinement through strategies such as sensory-informed practices, caregiver preparation, and clinician training, this protocol has strong potential to support more inclusive, timely, and effective hearing care for all children.

### 4.2 Implications for clinical practice and future research

The present study introduces a comprehensive assessment protocol that can be utilized for children undergoing speech-language or autism evaluations. Successful implementation of this protocol at other institutions depends on the establishment of referral pathways, workflows, and communication channels across multiple departments to ensure that requiring a hearing evaluation does not delay the developmental assessment workup. Careful consideration of staffing, scheduling availability, equipment, and personnel expertise should be addressed before implementing this protocol. Audiologists may require additional training on autism-related considerations, including common developmental and behavioral characteristics, referral pathways in their community, and strategies for discussing autism concerns with families. We recommend that audiologists have access to an autism expert to provide feedback on how to refine clinic workflows and navigate clinical management decisions for individual patients.

One opportunity to improve success rates with the protocol is the implementation of developmentally informed supports in the clinic that can be personalized to the unique needs and interest of a child. While pediatric audiologists at VUMC have extensive autism experience, standardized implementation of evidence-based supports for autistic children has not yet been established (30). In audiology clinics, transitions and learning new tasks can be facilitated with visual schedules, timers, sensory accommodations, and reinforcement materials personalized to a child's interests (31–33). Further research is needed to refine the protocol based on the required cognitive, language, social, and motor skills to perform specific tasks. For example, determining the specific skills required for a behavioral test method would allow appropriate selection and modification of testing procedures based on the child's abilities and interests. Additionally, knowledge of a child's developmental profile would assist in selecting appropriate, evidence-based, developmentally informed support strategies to optimize the testing session. Potential strategies could include sensory accommodations, transition supports, or behavior management techniques. Future research should focus on identifying effective strategies for systematically reducing sensitivity to transducers and probes, facilitating the collection of ear-specific data for autistic children. The consistent use of developmentally informed approaches is expected to improve data collection efficiency and reduce the time required to meet speech and discharge criteria.

Another area for clinical improvement involves identifying fast-track pathways for determining hearing status using physiological measures for autistic children. Of particular interest is the need to better understand the advantages and limitations of relying on DP OAEs when behavioral thresholds are unavailable or limited. In cases where children do not present with craniofacial anomalies or risk factors for delayed-onset reduced hearing, it may be appropriate to base discharge decisions on DP OAEs combined with measures of neural synchrony (e.g., MEMRs). Future refinement of the protocol presented here can be achieved by updating the DP OAE diagnostic criteria to reflect recently published normative data from infants and young children (34). Despite the potential utility of DP OAEs for evaluating hearing in autistic children, there is limited published research examining the rate of use, success, and clinical decision-making in this population. One unresolved question is whether autistic children exhibit a higher rate of middle-ear dysfunction, which serves as a contraindication for OAEs. Results from Yan et al. indicate a higher prevalence of PE tube use among autistic children (35). However, findings from the present study do not suggest a higher rate of middle-ear dysfunction in autistic children compared to the comparison group, when children with craniofacial differences are excluded. A second fast-track pathway warranting further investigation is sedated ABR testing. Clear guidance is needed for when to refer for sedated ABR testing rather than pursuing multiple behavioral testing sessions that are unsuccessful in determining hearing status (6). Evaluating these two potential mechanisms for fast-tracking assessment is expected to enhance the protocol and facilitate a timely and accurate diagnosis.

Lastly, the present study highlights the importance of considering social drivers of health in the timeliness of care access. We observed a high prevalence of public insurance use across our entire sample, likely reflecting both socioeconomic factors and medical complexity of the patient population. The higher proportion of public insurance within the group of autistic children (82.0% vs. 71.7%) likely stems from disability-based Medicaid eligibility pathways. Furthermore, half of the children in the dataset lived in neighborhoods classified as “Very Low” or “Low” opportunity according to the COI. Although the COI did not emerge as a significant covariate in our models, these neighborhood-level factors warrant continued consideration in clinical practice and future research. Previous studies have consistently demonstrated that children from low-opportunity neighborhoods experience delays in accessing health care and poorer health outcomes, including increased hospitalizations, higher prevalence of adverse medical conditions, and greater mortality rates (25). Extending these findings to hearing health care, it is probable that living in low-opportunity areas introduces systemic barriers to timely diagnosis and treatment, impacting both initial hearing evaluations and essential follow-up care.

When subsequent encounters are required, delays may be further compounded by barriers such as pre-authorization requirements, additional communication with providers, parental and child scheduling conflicts, and unreliable transportation. Collectively, these challenges exacerbate delays at every step of the diagnostic journey. Future research is needed to establish clinical guidelines that balance the necessity of comprehensive data with the need to minimize the time and cost burdens placed on families. For children with complex developmental profiles, modifications to clinic workflows could facilitate more sufficient data collection within a single encounter (e.g., extending appointment durations, multidisciplinary teams). Ultimately, these findings emphasize the importance of identifying and mitigating the specific access barriers faced by families in low-opportunity areas when seeking hearing health care for preschool- and early school-aged children.

### 4.3 Limitations

There are several limitations to the present study, particularly its reliance on retrospective clinical data from a single institution. Retrospective studies of clinical data are inherently susceptible to data omissions or inaccuracies within medical or billing records. Specifically, group membership misclassification is possible, as autism classification was determined based on ICD-10 diagnosis codes. Additionally, although the COI was prioritized to capture neighborhood-level opportunity, this metric may not account for insurance-specific barriers which may independently influence access. Another limitation is that the present study excluded children with craniofacial abnormalities, syndromes associated with reduced hearing, or those previously identified with permanent reduced hearing. This protocol is not appropriate for children with these characteristics who require more frequent monitoring and specialized management. Furthermore, as this sample was limited to children who were seen in the audiology clinic, our findings may not generalize to the broader population of children awaiting speech-language or autism assessment who bypass this testing.

The external validity of this study may be limited to medical centers with specialized pediatric care, including pediatric audiology services. Data were collected by multiple providers across various clinic locations. This study did not account for potential provider factors (e.g., experience level) or site-specific factors (e.g., appointment duration, booth size and setup), which may have influenced the likelihood of meeting discharge criteria or the type of assessment administered.

Another limitation of our protocol was the use of 25- and 20 dB HL cutoff for behavioral thresholds to meet speech and discharge criteria, respectively. These values are elevated relative to the 15 dB HL cutoff that is commonly recommended for pediatric hearing health care (36). Although conservative cutoffs are associated with stable classification over time (i.e., typical vs. reduced hearing), cases of “slight” reduced hearing (16 to 25 dB HL) will be missed (37–39). For example, results from a meta-analysis indicate that the prevalence of childhood reduced hearing is 13% for a criterion of >15 dB HL, and 2% for a criterion of >25 dB HL (38). Clinicians should carefully consider how their protocols affect the ability to identify cases of slight or mild reduced hearing. Children with slight or mild reduced hearing are more likely to have behavioral problems or reading difficulties compared to children who have typical hearing (40–43). Research is needed to establish evidence-based recommendations for the cutoff for typical hearing in autistic children, as any severity of reduced hearing likely compounds with existing developmental delays.

Although our statistical models controlled for age, we did not explicitly examine the interaction between age and autism classification. Furthermore, we did not attempt to characterize children's developmental profiles beyond the use of the ICD-10 diagnosis code for autism. As a result, we are unable to examine the effects of specific developmental skills on the effectiveness of the protocol. Moreover, the developmental profile of autistic children in our sample may not be representative of the broader population. At our institution, there are multiple departments that perform autism evaluations, and the referral rate for hearing evaluations varies across these clinics. Children with complex medical or developmental profiles, including those with co-occurring intellectual disabilities, may be underrepresented in our sample.

## 5 Conclusion

Using retrospective data from a single institution, this study demonstrates the clinical effectiveness of a standardized hearing assessment protocol for determining hearing status in 18-month to 8-year-old children. The protocol successfully enabled most children to meet criteria for progressing with speech-language or autism evaluations, yet key differences were observed based on autism classification. Autistic children were less likely to meet discharge criteria during the initial encounter, relied more heavily on physiological test methods, and required significantly more encounters to obtain sufficient hearing data. These findings highlight the unique challenges faced by autistic children during the diagnostic process and emphasize the need for tailored protocols to address these barriers. Future research should prioritize evaluating the effectiveness of the protocol in relation to children's developmental skills, exploring fast-track pathways for obtaining hearing data, and implementing the protocol at other sites. These efforts will be essential for advancing patient care and ensuring accurate and timely diagnosis of reduced hearing in autistic children.

## Data Availability

The data analyzed in this study is subject to the following licenses/restrictions: The datasets generated and analyzed during the current study are not publicly available due to restrictions related to patient confidentiality and institutional policies governing the use of clinical data. Requests to access these datasets should be directed to Angela Yarnell Bonino, angela.bonino@vumc.org.
